# Characterization of tri and tetra-nucleotide microsatellite loci for the freshwater snails *Promenetus exacuous* (Planorbidae) and *Valvata tricarinata* (Valvatidae) and their utility in population genetic studies

**DOI:** 10.1186/s13104-018-3301-1

**Published:** 2018-03-27

**Authors:** Patrick Yurco, Devon B. Keeney

**Affiliations:** Department of Biological and Environmental Sciences, Le Moyne College, 1419 Salt Springs Road, Syracuse, NY 13214-1301 USA

**Keywords:** Microsatellite loci, *Promenetus exacuous*, *Valvata tricarinata*, Population genetics, Gastropod genetic diversity

## Abstract

**Objective:**

*Promenetus exacuous* and *Valvata tricarinata* are freshwater snail species with widespread distributions throughout North America. Information regarding their genetic diversity and population connectivity are currently lacking. We utilized next generation sequencing to develop the first microsatellites for each species to investigate genetic diversity within and differentiation among populations.

**Results:**

Sixteen and seventeen microsatellite loci were developed for *P. exacuous* and *V. tricarinata*, respectively, and tested in a total of 43 *P. exacuous* and 48 *V. tricarinata* from two lakes approximately 183 km apart in New York State, USA. Fifteen *P. exacuous* loci were polymorphic in at least one lake and possessed 1–23 alleles and observed heterozygosities of 0.00–0.96 within individual lakes. Seventeen polymorphic *V. tricarinata* loci possessed 2–19 alleles and observed heterozygosities of 0.04–0.96 within lakes. Bayesian clustering using 12 loci for each species identified two distinct genetic populations, reflecting the two lakes. High assignment scores for individual snails to the lakes they were collected from supported strong population structure with minimal admixture at the scale of this study. These loci will be useful for investigating the genetic diversity and population structure of these species and indicate genetic differentiation may be common among their populations.

## Introduction

Despite their diversity, importance to ecosystems, and conservation concerns, basic taxonomic and biological information is lacking for many freshwater gastropods throughout North America [1, 2]. We report here the development of independent sets of 16 and 17 microsatellite loci for two understudied species of freshwater gastropods, *Promenetus exacuous* (Say, 1821) and *Valvata tricarinata* (Say, 1817). Microsatellites were developed for these particular species for future studies comparing their genetic connectivity throughout New York State based on the species’ disjunct and often sympatric populations throughout the region, their phylogenetic divergence and differing biological characteristics offering comparative insights into gastropod population connectivity throughout New York, and their current lack of genetic data and available microsatellite loci [3]. In addition, both species have widespread distributions throughout North America and secure global conservation status with localized areas of concern, potentially increasing the interest of these microsatellites to other researchers [4–6].

## Main text

### Materials and methods

*Valvata tricarinata* and *P. exacuous* were collected from Round Lake (43° 02′ 55.6″N, 75° 58′ 24.1″W) and Lake Saratoga (43° 03′ 12.6″N, 73° 43′ 11.8″W) using dip nets. Both lakes are in New York State and are approximately 183 km apart. To develop microsatellite loci, genomic DNA for next generation sequencing was extracted from the foot of a single *V. tricarinata* from Round Lake and a single *P. exacuous* from Lake Saratoga using a Qiagen Dneasy^®^ Tissue kit. DNA was eluted with 75 µl H_2_O and quantified using a NanoDrop 2000 (Thermo Scientific). Approximately 3 µg of RNA-free genomic DNA from each species was sent to the University of Wisconsin-Madison Biotechnology Center for DNA fragmentation and sequencing. Next generation sequencing was performed using an ION Torrent PGM system with each species allocated approximately 25% of a 318 chip. Raw genomic data were converted to FASTA format using Geneious ver. 8.1 [7]. Microsatellite motifs were identified using msatcommander 1.0.8 [8]. Parameters were set to search for perfect tri- and tetranucleotide repeats with a minimum length of eight repeat units to decrease the potential for genotyping errors due to stuttering common with dinucleotide repeats, and increase the likelihood of polymorphism [9, 10]. Primer %GC was set to 35–65%, optimal annealing temperature was 60 °C, and one primer from each pair was designed to have a 5′ CAG tag (CAGTCGGGCGTCATCA) (Table 1). Default settings were used for the remaining parameters.

**Table 1:** Microsatellite loci developed for (a) *Promenetus exacuous* and (b) *Valvata tricarinata*

Locus	Repeat motif	Primer sequence (5′–3′)	Label	Allele range (bp)	Round lake			Lake Saratoga		
					n	A	H_O_/H_E_	n	A	H_O_/H_E_
(a) *Promenetus exacuous*										
Pex216	(AAC)_11_	F: CAGTCGGGCGTCATCAACTTGGAATTGGCTGCCTC	VIC	239–278	24	4	0.50/0.53	14	6	0.57/0.79
		R: GCAAAGGCCGGATATTTCGATC								
Pex516	(AACT)_18_	F: CAGTCGGGCGTCATCACTGTCAGAAATACGCGGCTC	6-FAM	165–261	24	10	0.75/0.86	19	10	0.84/0.89
		R: GGCGCGAAATGGACTAACTG								
Pex577	(AAT)_10_	F: GCTGTCTTTCATGGTTCCGG	6-FAM	243–276	23	1	0.00/0.00	17	4	0.41/0.47
		R: CAGTCGGGCGTCATCAAATGTGTCTGCAGGCGTAC								
Pex757	(ACTC)_11_	F: TGAGAGCCCTTAAGTCGTGG	6-FAM	169–197	24	3	0.46/0.51	19	5	0.32/0.33
		R: CAGTCGGGCGTCATCAGTCAGCTACGTGATCTTGGC								
Pex1009	(AAC)_12_	F: TTATTGCCACTCACGTACGC	VIC	305–326	24	1	0.00/0.00	18	2	0.61/0.50
		R: CAGTCGGGCGTCATCATTAACGGTTCTGGCTTCCAC								
Pex1877	(AACT)_10_	F: CAGTCGGGCGTCATCAGCTTTGGAGTATGCTTGCC	NED	244–284	24	8	0.79/0.82	19	11	0.84/0.86
		R: CTAAGATTGGGAAGCCGCTG								
Pex2091	(AGAT)_16_	F: CAGTCGGGCGTCATCAGAGTGTTTCGGTGCCACAG	6-FAM	272–368	24	15	0.88/0.91	18	15	0.94/0.94
		R: AAATAGTGCCGAATGTGCCG								
Pex2117	(AACT)_9_	F: CAGTCGGGCGTCATCAACCTGCAAGAAAGACCTGC	VIC	192–208	22	3	0.36*/0.63	6	3	0.33/0.70
		R: CCTTTCCACCACATCACAGG								
Pex2181	(ACCT)_11_	F: CAGTCGGGCGTCATCAATGTAAGTGCGTGTGTAGCC	VIC	86–162	24	2	0.17/0.22	18	9	0.89/0.84
		R: ACTTCGCGTTTGTAGGTAGG								
Pex2263	(AAAG)_15_	F: CAGTCGGGCGTCATCAGCATCCATATTTCAAAGCTGGG	PET	158–226	24	8	0.83/0.82	19	11	0.89/0.89
		R: ACTGAAGTCCCTGAAGTGGC								
Pex2416	(ACT)_9_	F: CAGTCGGGCGTCATCATAGGGAGGCATACAAACGGAG	NED	314	24	1	0.00/0.00	19	1	0.00/0.00
		R: TTCGCTCAATACCCAGTGATC								
Pex2471	(ACC)_8_	F: AGGCAAACAGATGAGCTATGTC	PET	146–170	22	1	0.00/0.00	18	5	0.56/0.68
		R: CAGTCGGGCGTCATCATGGTACTGGGACTTCATGGC								
Pex2889	(AAAG)_8_	F: TACTGACTTGACGCCAATGC	PET	250–262	24	1	0.00/0.00	18	4	0.50/0.51
		R: CAGTCGGGCGTCATCAGTAGTCTAGGCCTTCGGTCC								
Pex2908	(ACCT)_17_	F: ACCTGCATGCCTAGCTACTG	NED	128–184	24	7	0.96/0.79	19	12	0.95/0.90
		R: CAGTCGGGCGTCATCATGTCATAAATCCGGCACTGC								
Pex2958	(AGAT)_15_	F: CAGTCGGGCGTCATCACTGCTATGGACGTGAGGGAG	PET	248–384	23	13	0.95/0.91	18	23	0.94/0.97
		R: TATTGATGGGCGGACGGATG								
Pex2972	(AACT)_12_	F: CAGTCGGGCGTCATCAGTCATCTACGCATGGGAAGC	PET	166–226	24	5	0.79/0.80	18	11	0.76/0.85
		R: GGCTTAAACTGGGACGATGC								
(b)* Valvata tricarinata*										
Vtr99	(AATC)_12_	F: CAGTCGGGCGTCATCACAGAGGTTCAAATCCCGGC	6-FAM	266–318	24	6	0.33/0.34	24	14	0.92/0.90
		R: AGTTGATCATCCCGCCGTAG								
Vtr115	(AGC)_8_	F: CTTTGCCTCTTCCGGACATG	NED	127–163	24	8	0.58/0.73	24	6	0.71/0.76
		R: CAGTCGGGCGTCATCACCTTCATTCCACCTCAGCAG								
Vtr565	(AAT)_25_	F: ACGGACTACAGGTGAATACAAC	6-FAM	186–270	23	7	0.65/0.85	23	19	0.96/0.95
		R: CAGTCGGGCGTCATCAGAAGTTCAATTTCGGCATGAG								
Vtr828	(AAC)_11_	F: CAGTCGGGCGTCATCATCTAGGGAAAGCGTGAGTGG	VIC	221–263	24	10	0.79/0.81	24	11	0.67/0.71
		R: GCCCACTACAACAAGCGAAG								
Vtr835	(ACT)_10_	F: TGTCAGATCACTCTTGGGCG	PET	203–224	24	2	0.08/0.08	24	5	0.42/0.59
		R: CAGTCGGGCGTCATCAACAACCTAGTGTGCCCTTC								
Vtr972	(AAG)_14_	F: CTCGTTTCCTGGCTGTTGTC	NED	127–169	24	7	0.67/0.61	24	7	0.67/0.76
		R: CAGTCGGGCGTCATCATAGAGTCCAAGTGTGAGGCG								
Vtr980	(AAG)_12_	F: ACGCTAAGCTTTGTACAGTGC	VIC	248–296	24	8	0.63/0.75	24	6	0.38/0.40
		R: CAGTCGGGCGTCATCAGAGTACCATCAAAGACGGCG								
Vtr1099	(ATC)_10_	F: CAGTCGGGCGTCATCATTCAGTGCAGACATTCGGG	VIC	258–279	24	3	0.58/0.55	24	5	0.46/0.52
		R: CTGCAGCCTCGTGAATTGAC								
Vtr1279	(AAT)_10_	F: CAGTCGGGCGTCATCAGCGAAGACAGAAATCCTCC	6-FAM	141–348	24	8	0.67/0.76	24	13	0.75/0.81
		R: ACAAATATATTTCGGTGCGCG								
Vtr1730	(AAG)_19_	F: CAGTCGGGCGTCATCATTGCTCCTTGGATTGGGATC	VIC	182–236	24	10	0.83/0.77	24	9	0.58*/0.76
		R: CAGCCCATTTCATCCTTGCC								
Vtr2328	(AAG)_10_	F: CCACAGGGCCAATAAATAACTG	NED	103–118	24	2	0.29/0.25	22	3	0.45/0.59
		R: CAGTCGGGCGTCATCATAGAGTCCAAGTGTGAGGCG								
Vtr2349	(AAAC)_14_	F: CAGTCGGGCGTCATCATGGGCACTGAAATCTCGTATG	VIC	324–354	24	4	0.29/0.27	13	6	0.23*/0.79
		R: CTTACGCCACTGCCACTAAC								
Vtr2388	(AAT)_25_	F: CAGGCCAAGATTCACACTGAC	PET	190–235	24	9	0.75/0.80	24	8	0.67/0.87
		R: CAGTCGGGCGTCATCAGTAACCCAGTCCGTGCTCG								
Vtr2492	(AAT)_20_	F: CAGTCGGGCGTCATCATCTCTGCCAGCTTACCACTG	NED	227–314	24	14	0.83/0.92	24	10	0.67/0.84
		R: GGACGTTGTGCTTCTATTCTCC								
Vtr2508	(AAT)_9_	F: CAGTCGGGCGTCATCATGTAGTGCCCATAGTCATGTAC	PET	117–132	24	2	0.50/0.42	20	2	0.20/0.39
		R: ACGCGTTCTCTTTAATACCTGC								
Vtr4154	(AAT)_9_	F: CAGTCGGGCGTCATCACCTACAGATCAGAGACGTACAC	PET	209–221	24	2	0.04/0.04	24	4	0.63/0.56
		R: TTGCAGATCAAGGTTGTCGC								
Vtr4287	(AGC)_8_	F: CAGTCGGGCGTCATCACCTTCATTCACCTCAGCAGC	NED	126–162	24	8	0.71/0.75	24	5	0.75/0.75
		R: CTTTGCCTCTTCCGGACATG								

Locus name is followed by repeat motif of sequenced allele, primer sequences, fluorescent dye label, total size range of alleles, and site-specific number of amplified individuals (*n*), number of alleles (A), and observed (H_O_)/expected (H_E_) heterozygosities. The 5′ primer tag is underlined and varied based on the 5′ beginning of the primer sequence. * Indicates statistically significant deviation from expected heterozygosity. GenBank Accession Numbers: *P. exacuous* MH000452–MH000467, *V. tricarinata* MH000435–MH000451

DNA was extracted from 24 *V. tricarinata* snails from each lake and 24 and 19 *P. exacuous* from Round Lake and Lake Saratoga, respectively. For each DNA extraction, the foot was removed and transferred to 400 µl of 5% Chelex containing 0.1 mg/ml proteinase K. The solution was incubated for approximately 8 h at 60 °C followed by eight minutes at 95 °C. DNA was utilized directly from these extractions. Microsatellite loci were amplified using three primer polymerase chain reactions (PCRs) [11] on individual loci with the Qiagen Type-it Microsatellite Kit. Each 10 µl reaction included 1X Type-it Multiplex PCR (Qiagen) reaction mix, 0.2 µM standard locus primer, 0.02 µM locus primer with CAG tag sequence, and 0.2 µM fluorescent-labeled CAG tag (PET, NED, 6-FAM, or VIC). The parameters of the PCRs were 5 min heat activation at 95 °C followed by 30 cycles of denaturation at 95 °C for 30 s, annealing for 90 s, and an extension at 72 °C for 30 s. The 30 cycles were followed by a final extension of 30 min at 60 °C. An initial round of PCRs was performed with a gradient of annealing temperatures ranging from 50 to 70 °C to determine optimal annealing temperatures. All optimized loci utilized an annealing temperature of 60 °C, except Pex1877 and Pex2263 (56 °C), and Pex 216 (53 °C). Genotypes were determined on an ABI 3730 × 1 96-capillary Genetic Analyzer at the DNA Analysis Facility at Yale University. PCR products from up to four loci utilizing different fluorescent dyes were combined in each well prior to submission. Alleles were scored using Geneious ver. 8.1 [7].

MICRO-CHECKER ver. 2.2 [12] was used to identify potential scoring errors from stuttering, large allele dropout, and/or the presence of null alleles. Alleles were analyzed for deviations from Hardy–Weinberg expectations within sites and overall linkage disequilibrium using Genepop on the Web [13, 14]. Significance tests with multiple comparisons used an adjusted critical value based on the B-Y False Discovery Rate (FDR) [15]. STRUCTURE ver. 2.4.3 [16] was used to determine if loci could infer population differentiation by using genotypes to assign individuals to genetic clusters and estimate the actual number of genetic populations using a Bayesian approach. A highly conservative subset of twelve loci for each species that did not display deviations from Hardy–Weinberg expectations in either site, did not include loci displaying linkage with each other, and that failed to amplify in no more than two snails in either population (*P. exacuous*: Pex577, Pex757, Pex1009, Pex1877, Pex2091, Pex2181, Pex2263, Pex2471, Pex2889, Pex2908, Pex2958, and Pex2972; *V. tricarinata*: Vtr99, Vtr565, Vtr828, Vtr835, Vtr980, Vtr1099, Vtr1279, Vtr2328, Vtr2388, Vtr2492, Vtr4154, and Vtr4287) were used for STRUCTURE analyses. STRUCTURE runs used an admixture model with five iterations, a burnin length of 100,000 and 100,000 steps in the Monte Carlo Markov Chain (MCMC). Separate runs for each species utilized LnPD as the selection criterion and the number of genetic populations (K) was allowed to range from 1 to 6.

### Results and discussion

Fifteen polymorphic *P. exacuous* loci possessed 1–23 alleles and observed heterozygosities ranged from 0.00 to 0.96 within individual lakes (Table 1). One locus deviated from Hardy–Weinberg expectations in Round Lake (Pex2117), potentially from null alleles and/or stuttering issues. Null alleles may also be present in Pex216 and Pex2117 in Lake Saratoga, with low amplification success likely prohibiting statistical significance. An additional sixteenth locus (Pex2416) was monomorphic in both populations, but is reported here as it may be polymorphic in other populations as observed between populations with several similar loci in the present study. Linkage disequilibrium was not detected between any pair of *P. exacuous* loci.

Seventeen polymorphic *V. tricarinata* loci possessed 2–19 alleles and observed heterozygosities of 0.04–0.96 within lakes (Table 1). Two loci deviated from Hardy–Weinberg expectations in Lake Saratoga (Vtr1730 and Vtr2349), potentially due to null alleles. Null alleles may also be present in Vtr2508 in Lake Saratoga. Linkage disequilibrium was detected among Vtr115, Vtr972, and Vtr4287.

**Fig. 1:**
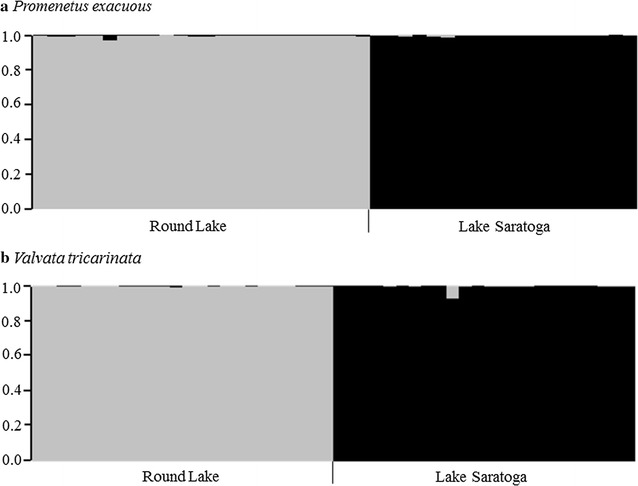
Results of STRUCTURE cluster membership analyses for **a** *Promenetus exacuous* and **b** *Valvata tricarinata* for K = 2. Vertical bars represent proportion of membership of individual snails for two genetic clusters (gray = Round Lake, black = Lake Saratoga). Labels below graphs indicate original sample sites

STRUCTURE results for both species supported two genetic populations (K = 2), reflecting the two sample locations (Fig. 1). All snails were assigned to the population they were sampled from with a high probability (97–100% for all snails except a single *V. tricarinata* from Lake Saratoga with 92%), revealing minimal admixture between these populations for both species (Fig. 1). The high assignment values reveal that these loci will be suitable for identifying gene flow patterns among populations experiencing varying levels of admixture. Multiple genetic groups were not detected within sites.

The development of microsatellites in these two understudied, distantly related species will enable researchers to examine the factors impacting the genetic diversity within and population structure among their populations, and gain additional insights into the biology, evolution, and conservation of freshwater gastropods. While our interests are primarily the dispersal and connectivity of these species throughout New York State and surrounding areas, these microsatellites may be used by other labs to address diverse questions in other regions. For example, although both species are globally secure, there are conservation concerns for specific populations throughout their range [4–6], and these markers may aid in conservation efforts. In addition, direct comparison of gastropods from different families over large geographic areas may reveal broad evolutionary dispersal patterns.

## Limitations

Due the potential for variation in regions flanking microsatellite loci and the relatively widespread distribution of both species, some of these loci may not amplify in populations throughout their range.

## Abbreviations

PCR: polymerase chain reaction
FDR: false discovery rate
K: number of genetic populations
NCBI: National Center for Biotechnology Information
*n*: number of amplified individuals
A: number of alleles
H_O_: observed heterozygosity
H_E_: expected heterozygosity
